# The long-term coronary heart disease risk of previously obese patients with type 2 diabetes mellitus

**DOI:** 10.1186/1472-6823-13-38

**Published:** 2013-10-03

**Authors:** Ritsuko Yamamoto-Honda, Hideki Ehara, Hiroji Kitazato, Yoshihiko Takahashi, Shoji Kawazu, Yasuo Akanuma, Mitsuhiko Noda

**Affiliations:** 1 Department of Diabetes and Metabolic Medicine and Diabetes Research Center, National Center for Global Medicine, 1-21-1 Toyama, Shinjuku-ku, Tokyo 162-8655, Japan; 2 The Institute for Adult Diseases, Asahi Life Foundation, 2-2-6, Nihonbashibakurouchou, Chuou-ku, Tokyo 103-0002, Japan; 3 Ehara Medical Clinic, 1-10 Shouwa-cho, Tuyama-city, Okayama 708-0886, Japan; 4 Department of Diabetes and Endocrinology, Oomori Red Cross Hospital, 4-30-11 Chuo, Oota-ku, Tokyo 143-8527, Japan; 5 Division of Diabetes and Metabolism, Iwate Medical University School of Medicine, 19-1 Uchimaru, Morioka, Iwate 020-8505, Japan

**Keywords:** Diabetes, Previous obesity, Coronary heart disease

## Abstract

**Background:**

Obesity is associated with insulin resistance, development of diabetes, and coronary heart disease. There is limited information on the contribution of previous obesity on the risk of coronary heart disease. We aimed to examine the effect of previous history of obesity on the occurrence of coronary heart disease in patients with diabetes.

**Methods:**

We carried out a retrospective chart analysis of 315 type 2 diabetic patients without obesity and without atherosclerotic cardiovascular events at their initial hospital visit (men/women 236/79; mean ± standard deviation; age 53.1 ± 6.6 years; maximal body mass index before enrollment (MAXBMI) 26.6 ± 3.4 kg/m^2^; decrease of the BMI at enrollment from MAXBMI (deltaBMI) 4.23 ± 2.62 kg/m^2^) to investigate the association of previous obesity (MAXBMI larger than 30 kg/m^2^) with the long-term incidence of cardiovascular events. Of 315 patients, forty-eight were previously obese.

**Results:**

After median follow-up of 13.9 years, 48 patients developed coronary heart disease. The Kaplan-Meier analysis exhibited that coronary heart disease occurred more frequently in previously obese patients than in subjects in the reference category (22 kg/m2 < or = MAXBMI < 25 kg/m^2^) and that the effect lasted proportionally over follow-up periods. Multivariate Cox regression models showed that hazard ratios and corresponding 95% confidence intervals of coronary heart disease for patients with previous obesity compared with subjects in the reference category were 2.52 and 1.15 to 5.50 (p value = 0.020) after adjustment for age, sex, smoking status, systolic blood pressure, total cholesterol and HDL cholesterol. In this cohort, deltaBMI strongly correlated with MAXBMI and also behaved as a risk factor. The hazard ratios and 95% confidence intervals by the increment of one standard deviation of deltaBMI after adjustment for age, sex, smoking status, systolic blood pressure, total cholesterol and HDL cholesterol were 1.38 and 1.08 to 1.79 (p value = 0.013).

**Conclusions:**

Previous obesity and/or large body weight loss before admission might act as an increased risk for coronary heart disease.

## Background

The accelerated Westernization of lifestyles has led to a rapid increase in the number of type 2 diabetic patients worldwide, to the extent that diabetes has now been recognized as a threat to public health. In patients with type 2 diabetes (T2DM), a high prevalence of cardiovascular diseases is observed at a relatively young age [1]. Thus, risk factors for atherosclerosis must be evaluated in patients with T2DM.

Obesity is frequently associated with insulin resistance, development of diabetes [2,3], and atherosclerotic cardiovascular disease [1,4,5]. The body mass index (BMI) of Japanese has been reported to increase until the age group of 60-69 [6,7] and a BMI larger than 27.5 was associated with an increased risk of myocardial infarction [8].

In patients with T2DM, past obesity might be overlooked because those who had previously been overweight/obese were often not so thereafter [9]. This might be a part of the reason why subjects classed as normal-weight at the time of incident diabetes showed non-significant but higher rates of cardiovascular mortality than subjects who were classed as overweight/obese at the time of incident diabetes [10].

Previously obese diabetic patients were likely to be associated with higher burden of atherosclerosis [11,12], but the long-term risk comparison of coronary heart disease between previously obese diabetic patients and never obese diabetic patients has not been examined in detail. The aim of the present study was to examine the effect of overweight/obesity before their first visit to a hospital on the long-term occurrence of coronary heart disease in patients with T2DM.

## Methods

### Population for analysis

This study was a part of the retrospective cohort follow-up study of T2DM. A total of 560 subjects with the diagnosis of T2DM visited a hospital located in the center of Tokyo between 1987 and 1992 [13,14]. Of the 560 subjects, we selected 430 subjects (age under 65 years). We excluded patients aged over 65 years in the present analysis because about one-third of the patients in this age group had already experienced cardiovascular events at their first visit to the hospital. Of the 430 subjects, an additional 61 subjects were excluded from the present analyses because of the poor quality of their medical records, leaving 369 subjects. We next excluded another 49 subjects with coronary heart disease or stroke at the time of their first visit to the hospital, leaving 320 subjects. We finally selected 315 non-obese subjects (236 men and 79 women) for enrollment in the present analysis. Since their first visit to the hospital, the patients were encouraged to reduce and maintain their BMI at a value under 22 kg/m^2,^ to walk 10,000 steps a day, and to consume a low-fat (less than 30% of the daily caloric intake), low-energy (25 – 27 kcal/ideal body weight/day) diet. Intake ratios of saturated fatty acids, monosaturated fatty acids, and polyunsaturated fatty acids of 3: 4: 3 were recommended.

### Study variables

We retrospectively evaluated the charts of the 315 patients from the first visit to the hospital (the study’s starting point) to the occurrence of coronary heart disease (the study’s endpoint) or January 2004, whichever came first.

We obtained the patients’ medical records including smoking status (never smoker, ex-smoker or smoker at enrollment) and their weight history (self-reported maximal body weight and the age at which patients reached their maximal body weight) and examined their BMI, systolic and diastolic blood pressure, serum total cholesterol levels (determined enzymatically), triglyceride levels (determined enzymatically), HDL-cholesterol level (determined using the deposition method), and glycosylated hemoglobin (HbA1c) level. MAXBMI (maximal body mass index before enrollment) was calculated as: [self-reported maximal weight before enrollment (kg)]/[height at enrollment (m)]^2^. The HbA1c level was determined using high-performance liquid chromatography using HLC-723GHb II (Tosoh, Tokyo) at enrollment. The HbA1c values measured using HLC-723GHb II were calibrated to a National Glycohemoglobin Standardization Program value (%) [15]. The LDL-cholesterol level was calculated according to the Friedewald equation [16]. The diagnosis of coronary heart disease (coronary insufficiency and myocardial infarction) was made according to criteria defined by the Framingham Heart Study [17]. The diagnosis of myocardial infarction was determined by specified electrocardiographic changes accompanied by an elevation of serum enzymes. Coronary insufficiency was defined as prolonged ischemic chest pain accompanied by transient ischemic abnormalities on electrocardiography. Coronary angiography was performed in all patients of suspicious coronary heart disease and a diagnosis of coronary heart disease was confirmed by narrowed or blocked coronary arteries. This research program conformed to the ethical recommendations for epidemiologic studies, as declared by the Ministry of Health, Labour and Welfare in Japan, and was approved by the National Center for Global Health and Medicine Research Ethics Committee.

### Statistical analysis

The data analysis was performed using R [18]. The continuous variables were summarized as mean ± standard deviation or median with 25th-75th percentiles (for variables not showing normal distribution). For analysis, we grouped MAXBMI as follows: 18.5 to less than 22, 22 to less than 25 (reference category), 25 to less than 27.5, 27.5 to less than 30, and 30 or greater. There were no patients whose MAXBMI was less than 18.5. The Kaplan-Meier method was used to estimate survival curves of cardiovascular events associated with the MAXBMI category. The log-rank test was used to compare the unadjusted survival curves. Multivariate Cox regression models were used to examine the interaction of known risk factors. P-values less than 0.05 were considered statistically significant.

## Results

### Patients

Mean age ± standard deviation of the 315 patients was 53.1 ± 6.6 years, mean MAXBMI ± standard deviation was 26.6 ± 3.4 kg/m^2^, and mean BMI at enrollment ± standard deviation was 22.4 ± 2.7 kg/m^2^.

Among the 315 patients who had no history of cardiovascular events at the start point, 48 of them developed coronary heart disease. Twenty-seven patients died without experiencing coronary heart disease and 168 were continuing to visit the hospital without having experienced coronary heart disease. Seventy-two patients had stopped visiting the hospital before January 2004 without having experienced coronary heart disease. The median observation period from the start of the observation to the endpoint was 13.9 years. The characteristics of the 315 patients at the start of the observation period are summarized in Table 1. Forty-eight had history of obesity. Ten of them had become obese before the age of thirty and another twenty of them had become obese before the age of forty.

**Table 1:** Mean values or prevalence of factors at baseline of the 319 patients

	**MAXBMI, kg/m**^**2**^				
	**Less than 22.0**	**22.0 to less than 25.0 (reference)**	**25.0 to less than 27.5**	**27.5 to less than 30**	**30.0 or greater**
Number of patients (men/women)	22 (15/7)	84 (63/21)	95 (80/15)	66 (48/18)	48 (31/17)
Age (years)	52.4 ± 5.6	53.4 ± 7.5	52.8 ± 6.3	53.5 ± 6.7	53.3 ± 6.2
Observation period (years)	14.5 ± 3.6	12.6 ± 5.4	12.3 ± 5.9	11.9 ± 5.6	11.4 ± 5.1
Ex/present smokers (%)	22.7/45.5	28.6/27.4	30.5/40.0	36.3/33.3	14.6/39.6
MAXBMI (kg/m^2^)	21.0 ± 0.86§	23.7 ± 0.77	26.2 ± 0.65§	28.5 ± 0.66§	32.6 ± 2.12§
DeltaBMI (kg/m^2^)	2.17 ± 1.38§	3.21 ± 1.68	3.47 ± 1.81	4.89 ± 2.13§	7.63 ± 3.13§
Time interval between MAXBMI and BMI at enrollment (years)	8.5 (4–14.5)	15 (6–15)	8.5 (4–15) †	9 (3.5-16.5)*	15 (10–22)
BMI at enrollment (kg/m^2^)	18.9 ± 1.7*	20.5 ± 1.7	22.8 ± 1.9§	23.6 ± 2.2§	25.0 ± 2.7§
HbA1c (%)	9.2 ± 1.8	9.2 ± 2.0	9.0 ± 2.1	9.8 ± 2.0	9.9 ± 2.1
Medications for hyperglycemia					
OHA (%)	45.5	40.5	49.5	42.4	47.9
Insulin (%)	22.7	11.9	8.4	12.1	14.6
Total cholesterol (mmol/L)	5.54 ± 0.86	5.37 ± 1.33	5.23 ± 0.99	5.60 ± 1.14	5.60 ± 1.18
HDL cholesterol (mmol/L)	1.47 ± 0.45*	1.20 ± 0.34	1.14 ± 0.30	1.11 ± 0.26	1.10 ± 0.28
LDL cholesterol (mmol/L)	3.52 ± 0.88	3.49 ± 1.28	3.24 ± 0.92	3.64 ± 1.03	3.57 ± 0.97
Triglyceride (mmol/L)	1.34 ± 0.66	1.81 ± 1.78	2.06 ± 1.54	2.07 ± 1.40	2.24 ± 1.59
Systolic blood pressure (mmHg)	130.9 ± 18.5	128.0 ± 20.1	128.9 ± 17.4	131.2 ± 17.9	132.6 ± 18.5
Diastolic blood pressure (mmHg)	77.0 ± 10.1	75.7 ± 12.1	79.3 ± 10.7	80.2 ± 9.7	78.7 ± 12.6
Medications for hypertension (%)	0.0	6.0	17.9	12.1	8.3
Diabetic retinopathy (%)	22.7	27.7	20.0	36.4	50.0§

Values are presented as a percentage (%), as means ± SD, or as median with 25th-75th percentiles; * P < 0.05, † P < 0.01, § P < 0.001 versus the reference category.

*Abbreviations:BMI*, body mass index; *MAXBMI*, maximal body mass index before enrollment; *deltaBMI*, decrease of the BMI at enrollment from MAXBMI.

At enrollment, patients with a MAXBMI greater than or equal to 30 kg/m^2^ had a larger BMI than patients with a MAXBMI less than 30 kg/m^2^. Patients with a MAXBMI greater than or equal to 30 kg/m^2^ had higher HbA1c and lower HDL cholesterol values, but the difference was not statistically significant. Patients with a MAXBMI greater than or equal to 30 kg/m^2^ had a higher incidence of diabetic retinopathy than patients with a MAXBMI less than 30 kg/m^2^. This result was consistent with the report of Ogawa et al. [11].

### The Kaplan-Meier analysis

**Figure 1:**
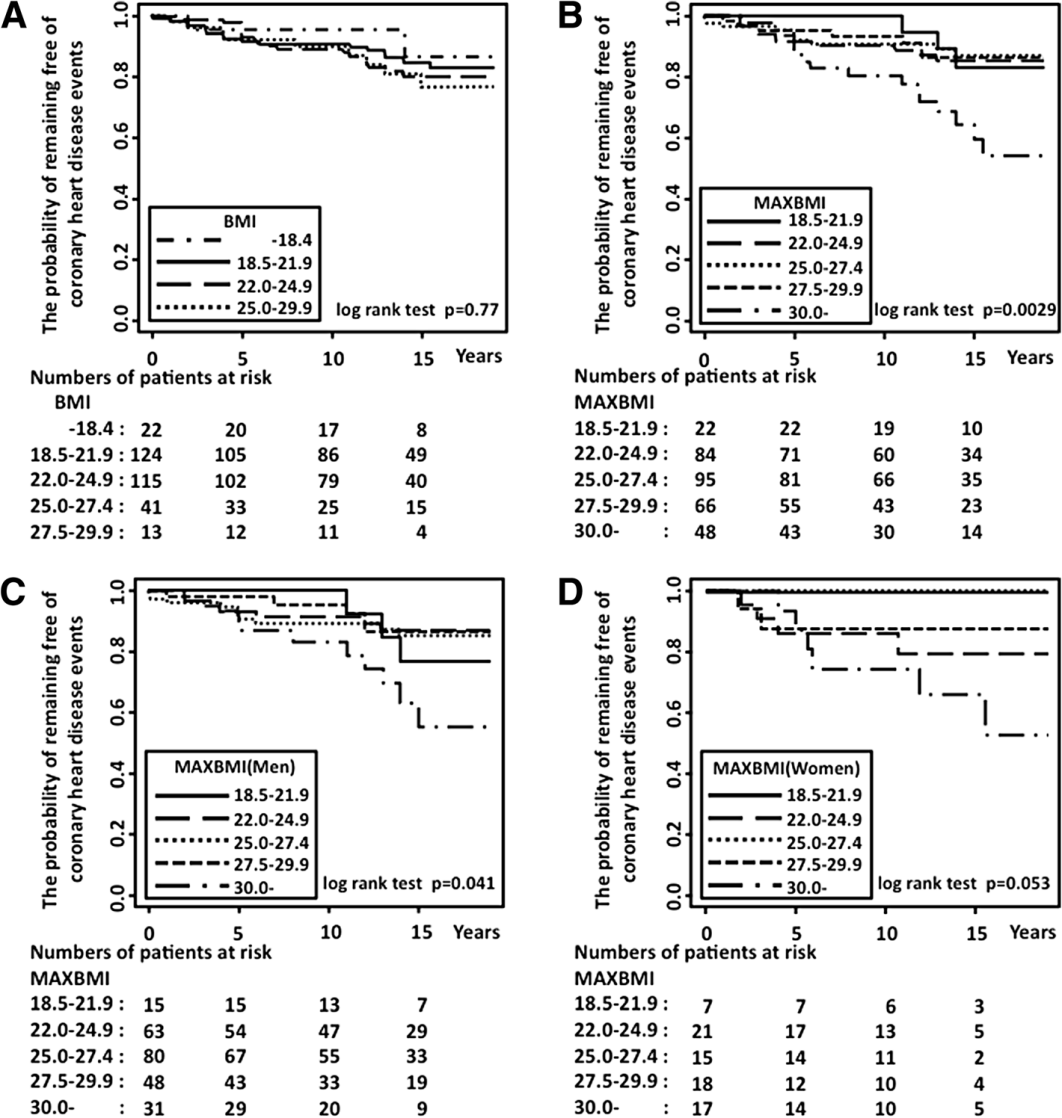
**Kaplan-Meier survival curves from the probability of remaining free of coronary heart disease events. A**: categorized by the body mass index (BMI) at enrollment, **B**: categorized by the maximal body mass index before enrollment (MAXBMI), **C**: categorized by the maximal body mass index before enrollment (MAXBMI) with diabetic men, **D**: categorized by the maximal body mass index before enrollment (MAXBMI) with diabetic women.

The Kaplan-Meier analysis exhibited that coronary heart disease occurred more frequently in previously obese patients and that the effect seemed proportional over the follow-up periods (Figure 1B; MAXBMI compared with subjects in the reference category for the log-rank statistic; p = 0.0029). After stratification by gender, coronary heart disease occurred more frequently both in men and in women, but the results with women did not reach significance (Figure 1C and 1D). The occurrence of coronary heart disease of previously overweight patients was comparable to that of never-overweight patients (Figure 1B). In addition, the occurrence of coronary heart disease was not affected by BMI category at enrollment (Figure 1A).

### Cox regression models

Next we used Cox regression models to examine the interaction of known risk factors as confounding factors with the incidence of cardiovascular events. Calculation by multivariate Cox regression models exhibited a presumable threshold effect rather than a graded increase of MAXBMI, suggesting unrecognized factors acted with regard to previous obesity. Hazard ratios and corresponding 95% confidence intervals of coronary heart disease for patients with previous obesity compared with subjects in the reference category (22 < or = MAXBMI < 25) were 2.52 and 1.15 to 5.50 (p value = 0.020) after adjustment for age and sex with additional adjustment for smoking status, serum lipids, and blood pressure (Table 2).

**Table 2:** Cox proportional hazards regression analysis with maximal body mass index before enrollment

	**Maximal body mass index before enrollment (MAXBMI, kg/m**^**2**^**)**				
	**Less than 22.0**	**22.0 - 24.9 (reference)**	**25.0 - 27.4**	**27.5 – 29.9**	**30.0 or greater**
Model 1 HR (95% CI)	0.90 (0.25-3.24) P = 0.87	1.00	0.85 (0.36-2.01) P = 0.70	0.98 (0.39-2.44) P = 0.96	2.78 (1.28-6.01) P = 0.009
Model 2 HR (95% CI)	1.07 (0.29-4.00) P = 0.92	1.00	0.90 (0.38-2.17) P = 0.82	0.96 (0.39-2.42) P = 0.94	2.52 (1.15-5.50) P = 0.020
Model 3 HR (95% CI)	1.00 (0.27-3.73) P = 0.99	1.00	0.94 (0.39-2.24) P = 0.88	0.94 (0.37-2.36) P = 0.90	2.31 (1.05-5.08) P = 0.038

Analysis was performed to estimate hazard ratios (HR) with corresponding 95% confidence intervals (95% CI) for coronary heart disease associated with maximal body mass index before enrollment (MAXBMI).

Reference categories were subjects with a MAXBMI between 22.0 and 24.9.

Model 1 is adjusted for age and sex; model 2, model 1 plus smoking status, systolic blood pressure, total cholesterol and HDL cholesterol; model 3, model 2 plus HbA1c values at enrollment.

In the present cohort, a larger decrease of BMI before enrollment was observed in patients with larger MAXBMI (Table 1). The differences between BMI at enrollment and MAXBMI (deltaBMI) strongly correlated with MAXBMI values (Pearson’s correlation coefficient = 0.64) and deltaBMI also related with the occurrence of coronary heart disease. The hazard ratios by the increment of one standard deviation of deltaBMI (2.62 kg/m^2^) were calculated as 1.38 (95% confidence intervals: 1.08 to 1.79; p value = 0.013) after adjustment for age, sex, smoking status, serum lipids, and blood pressure (Table 3). The association became weak after adjustment for HbA1c at enrollment (Table 3), suggesting that prolonged poor glycemic control was in part related to the patients with a large deltaBMI.

**Table 3:** Cox proportional hazards regression analysis with differences between BMI at enrollment and MAXBMI (deltaBMI)

Model 1	
HR (95% CI) per 1 SD	1.35 (1.03 - 1.72) P = 0.029
Model 2	
HR (95% CI) per 1 SD	1.38 (1.08 - 1.79) P = 0.013
Model 3	
HR (95% CI) per 1 SD	1.31 (0.99 – 1.70) P = 0.057

Analysis was performed to estimate the hazard ratios (HR) with the corresponding 95% confidence intervals (95% CI) for coronary heart disease associated with the differences between BMI at enrollment and MAXBMI (deltaBMI).

Model 1 is adjusted for age and sex; model 2, model 1 plus smoking status, systolic blood pressure, total cholesterol and HDL cholesterol; model 3, model 2 plus HbA1c values at enrollment.

## Discussion

In the present cohort where deltaBMI strongly correlated with MAXBMI, we could not distinguish which of the two, namely a threshold value of previous obesity or a graded relationship of preceding weight loss before enrollment, behaved as principal risk factor. In order to find out which of the two (or both) behaved as a principal risk factor, other cohorts in which there were weaker correlation between deltaBMI and MAXBMI should be examined.

Obesity exacerbated atherosclerosis before enrollment by way of the classical cardiovascular risk factors and other factors such as high serum levels of lipoprotein (a), increased oxidative stress, and low-grade inflammation [19,20]. Accelerated atherosclerosis was more often observed in previously obese T2DM patients than in never-obese T2DM patients [11,12]. With the best possible medical care including the control of blood pressure and serum LDL cholesterol levels after enrollment, atherosclerosis progressed to coronary heart disease in previously obese patients. Factors such as persistent dysfunction of HDL including cholesterol efflux capacity, and persistent low-grade inflammation might play roles to progression of atherosclerosis even after weight reduction [21-24]. The degree of visceral fat reduction was well correlated with alteration of both inflammatory and anti-inflammatory cytokine levels, while the degree of visceral fat reduction in response to weight reduction varied from patient to patient [25].

A large amount of weight loss due to persistent hyperglycemia or accelerated atherosclerosis [26] induced sarcopenia with reduction of gynoid fat [27-29], resulting in features of so-called “normal weight obesity” or “metabolically obese individual” at enrollment [30]. Elevated risks for coronary heart disease were also observed with short-term, unintentional weight loss in middle adulthood [31].

We admit that the present study has several additional limitations. The most important limitation is that we do not have data about waist circumferences, body fat content, daily physical activity and inflammatory parameters such as CRP, fibrinogen, or proinflammatory cytokines of the participants at enrollment, nor could we examine the result of body weight loss on body composition of the participants. Second, the patients recruited in the study were from a single hospital in the center of Tokyo and many of the patients were businesspersons. After admission, blood glucose, blood pressure and serum LDL cholesterol levels were under control by medication if necessary, so that the effect of obesity on these risk factors might be cancelled. Thus, replication studies including other cohorts living under different life styles with a different treatment strategy of diabetes are warranted. Third, due to the small numbers (only one-quarter of the sample, 78 cases) of women patients at enrollment, we could not conclude the risk of “previously obese” diabetic women. Finally, survival biases might play a role. Patients classed as normal-weight at the time of incident diabetes were reported to show higher rates of overall mortality than subjects who were classed as overweight/obese at the time of incident diabetes [10].

## Conclusions

Obesity before enrollment and/or large body weight loss before the admission acted as a life-long risk factor for coronary heart disease in patients with T2DM. In clinical practice, it is advisable to ask patients with T2DM what their previous maximal body weight was, and to calculate their MAXBMI and deltaBMI to estimate cardiovascular risk. From the epidemiological point of view, it is advisable to avoid obesity in young adults to reduce the risk of developing diabetes [32,33] as well as to reduce the risk for coronary heart disease [34,35]. It is also advisable to advise patients to undergo a medical check-up every year so as not to leave diabetes undiagnosed, untreated, or left poorly controlled.

## Abbreviations

T2DM: Type 2 diabetes; BMI: Body mass index; MAXBMI: Maximal body mass index before enrollment; DeltaBMI: Decrease of the BMI at enrollment from MAXBMI.

## Competing interests

The authors declare that they have no competing interests.

## Authors’ contributions

RY-H participated in the design of the analysis on MAXBMI, performed the statistical analysis, and wrote the paper. HE conceived of the study and collected the data. HK conceived of the study and set up the cohort. YT contributed the statistical analysis. SK was involved in drafting the manuscript. YA examined the participants of the cohort as a physician and conceived of the study. MN conceived of the study and helped to draft the manuscript. All authors have read and approved the final manuscript.

## Pre-publication history

The pre-publication history for this paper can be accessed here:

http://www.biomedcentral.com/1472-6823/13/38/prepub
